# Moderate to severe chronic arteriolar lesions is an independent risk factor for adverse renal outcomes in IgA nephropathy

**DOI:** 10.1371/journal.pone.0320635

**Published:** 2025-04-24

**Authors:** Qian Wu, Yi Chen, Miaoying Shen, Yuyuan Cai, Haokai Yu, Lei Zhou, Haifeng Yang, Chuan Zou

**Affiliations:** 1 Second Clinical Medical College, Guangzhou University of Chinese Medicine, Guangzhou, Guangdong, China; 2 School of Mathematics and Physics, Southwest University of Science and Technology, Mianyang, Sichuan, China; 3 Department of Pathology, Guangdong Provincial Hospital of Chinese Medicine, The Second Affiliated Hospital of Guangzhou University of Chinese Medicine, Guangzhou, Guangdong, China; 4 Department of Nephrology, Guangdong Provincial Hospital of Chinese Medicine, The Second Affiliated Hospital of Guangzhou University of Chinese Medicine, Guangzhou, Guangdong, China; Phramongkutklao College of Medicine, THAILAND

## Abstract

**Background:**

The impact of chronic arteriolar lesions on the prognosis of IgA nephropathy remains controversial. This study aims to explore the value of chronic arteriolar lesions of varying degrees in predicting the prognosis of IgA nephropathy patients and analyze the associated risk factors that contribute to the formation.

**Methods:**

A retrospective analysis was conducted on 853 patients diagnosed with IgA nephropathy through renal biopsy at Guangdong Provincial Hospital of Traditional Chinese Medicine between September 1, 2005, and December 31, 2021. Eventually, a total of 574 cases were included in this study. According to the degree of chronic arteriolar lesions, the patients were divided into four groups: no lesion group (n=115), mild lesion group (n=287), moderate lesion group (n=131), and severe lesion group (n=41). Relevant clinical and pathological features and renal outcomes were recorded. Kaplan-Meier analysis, Cox proportional hazards regression, and receiver operating characteristic (ROC) curve analysis were utilized to examine the relationship between different degrees of chronic arteriolar lesions and the prognosis of IgA nephropathy. Additionally, risk factors associated with the development of moderate to severe chronic arteriolar lesions were identified.

**Results:**

Worse clinical and pathological features were observed in the moderate to severe lesions group (P<0.05). Moderate to severe chronic arteriolar lesions (aHR=3.357, 95%CI: 1.018–11.071, P=0.047), creatinine, S1, E1, T2, and C2 were identified as independent risk factors for adverse renal outcomes. Cox multivariate regression analysis on moderate to severe chronic arteriolar lesions demonstrated that creatinine, T2, and C2 were independent risk factors for adverse renal outcomes in patients with moderate to severe chronic arteriolar lesions.

**Conclusion:**

Moderate to severe chronic arteriolar lesions independently increases the risk of adverse renal outcomes.

## Introduction

IgA nephropathy (IgAN) is a primary glomerulonephritis characterized by the deposition of immunoglobulin A (IgA) in the mesangial area of the renal glomerulus [1]. Approximately 20%-40% of IgAN patients progress to end-stage renal disease (ESRD) within 20 years [2]. Previous studies have indicated that factors such as urinary protein, renal function, and blood pressure are linked to IgAN progression. Additionally, kidney pathological changes play a significant role as independent risk factors for disease progression [3], Studies have confirmed mesangial hypercellularity (M), segmental glomerulosclerosis (S), and tubular atrophy/interstitial fibrosis (T) lesions as independent risk factors for poor renal prognosis, In particular, T lesions are considered the strongest predictor of renal survival [4], underscoring the importance of pathology analysis.

Currently, most research on prognostic factors related to IgAN pathology focuses on aspects such as crescents, glomerulosclerosis, and interstitial fibrosis. Studies on vascular changes primarily concentrate on microvascular lesions [5,6], with relatively limited attention given to chronic arteriolar lesions. Nevertheless, chronic arteriolar lesions is highly prevalent in the histopathological lesions of IgAN. In a retrospective study of IgAN patients, the prevalence of arteriolar lesions was reported to be as high as 54.6%, encompassing arterial wall thickening (30.7%) and hyaline arteriolosclerosis (23.9%) [7]. The original Oxford classification cohort also incorporated two pathological indicators, namely arterial intimal thickening and arterial hyalinosis. However, these vascular lesions were excluded from the Oxford classification [8] due to their lack of statistically significant correlation with the endpoint.This suggests that vascular lesions show heterogeneity in the prognosis of IgAN patients. Recent research has established a notable association between arteriolar lesions and poor renal survival in IgAN patients [9]. However, these studies have mainly focused on the presence or absence of chronic arteriolar lesions or specific lesions like arteriolar hyalinosis. Some studies [7] have employed histological classification methods designed for early IgAN, including the Lee classification [10] and Katafuchi semi-quantitative score [11], to assess chronic arteriolar lesions severity. These studies have demonstrated an association between the degree of chronic arteriolar lesions and prognosis. Nevertheless these grading methods for vascular lesions are cumbersome and possess limited practical applicability in clinical settings. In clinical experience, we have noticed that when there is concomitant intimal thickening along with arteriolar hyalinosis, the clinical prognosis tends to be more severe for IgAN patients. Consequently, This study developed a novel classification method to assess the severity of chronic arteriolar lesions, based on the vascular lesion assessment criteria from the 2009 Oxford Classification and the Banff 97 grading schema [8,12], while incorporating findings from prior research on arteriolar lesion classification [,^7^,^9^,^13^]. This method categorizes chronic arteriolar lesions into mild, moderate, and severe levels. Utilizing this classification, the study aimed to investigate the association between varying degrees of chronic arteriolar lesions and the prognosis of IgA nephropathy (IgAN). Additionally, the study analyzed risk factors that contribute to the progression of chronic arteriolar lesions.

## Materials and methods

### Ethics statement

Analysis was conducted using anonymized data accessed on June 08, 2023, ensuring that only de-identified data were utilized for the study.This study has been approved by the Ethics Committee of Guangdong Provincial Hospital of Traditional Chinese Medicine (Approval No. YE2022-181-01), which is in line with the ethical principles of clinical research as well as the Declaration of Helsinki and International Ethical Guidelines for Research Involving Human Health, among other documents. The ethics committee waived informed consent because of the retrospective nature of the study.

### Patients

This retrospective review encompassed a total of 853 patients diagnosed with primary IgAN following renal biopsy at Guangdong Provincial Hospital of Traditional Chinese Medicine between September 1, 2005, and December 31, 2021.

Inclusion criteria: (1) Age ≥ 18 years old;(2)A definitive diagnosis of IgAN was confirmed through a kidney biopsy.

Exclusion criteria: Patients with fewer than 8 glomeruli on biopsy. Patients with incomplete clinical, renal pathology, or follow-up data. Patients who reached end-stage renal disease (ESRD) with an estimated glomerular filtration rate (eGFR) less than 15 ml/min/1.73m^2^. Renal biopsy revealing microvascular lesions, including endothelial swelling, mucoid degeneration, fibrinoid necrosis of vascular tufts, and microthrombi formation in small renal arteries. Follow-up time less than 12 months with no occurrence of endpoint events. Presence of systemic diseases such as Henoch-Schonlein purpura nephritis, cirrhosis, etc., which can lead to secondary renal deposition.

**Selection of Covariates:** The covariates included in this study were selected based on variables previously demonstrated to be associated with the progression of renal vascular lesions in prior studies, as well as variables that showed a significant correlation with varying degrees of chronic arteriolar lesions in the current study (p < 0.05). The inclusion of these covariates was aimed at comprehensively adjusting for potential confounders, thereby enhancing the accuracy and reliability of the research findings.
**Clinical data:** Baseline data collected during biopsy encompassed the following clinical and laboratory parameters: Age, gender, medical history, systolic blood pressure (SBP), diastolic blood pressure (DBP), mean arterial pressure (MAP), urinary protein quantification, urinary red blood cell count (URBC), blood urea nitrogen (BUN), serum creatinine (Scr), estimated glomerular filtration rate (eGFR) calculated using the CKD-EPI equation [14], uric acid (UA), serum albumin (Alb), hemoglobin (Hb), total cholesterol (TC), triglycerides (TG), high-density lipoprotein cholesterol (HDL-C), low-density lipoprotein cholesterol (LDL-C), fibrinogen (FIB), serum IgA and C3, and treatment information.

### Pathological data

Renal pathology specimens were evaluated using immunofluorescence, optical microscopy. Pathological characteristics of chronic arteriolar lesions were assessed utilizing Hematoxylin and Eosin (H&E) staining, Masson’s trichrome staining, Periodic Acid-Schiff (PAS) staining, and Periodic Acid-Silver Methenamine (PASM) staining. Immunofluorescence staining was employed on renal tissue sections to quantify the intensity of glomerular deposits of IgA and C3. The intensities of mesangial IgA and C3 deposits were classified as negative (-), trace (+/-), weak (+), moderate (++), strong (+++), or very strong (++++) based on their staining patterns.Renal biopsy tissues were reviewed by two experienced pathologists and nephrologists according to the Oxford IgAN classification (MEST-C scoring) [4]: Mesangial cell proliferation was categorized as scoring ≤0.5 (M0) or >0.5 (M1). Endocapillary cell proliferation was classified as either absent (E0) or present (E1). Segmental glomerulosclerosis was characterized as either absent (S0) or present (S1). The extent of tubular atrophy/interstitial fibrosis in the kidney was categorized as ≤25% (T0), 25–50% (T1), or > 50% (T2). Crescent formation was classified as absent (C0), present in < 25% glomerulus (C1), or present in ≥25% of glomeruli (C2).Systematically re-evaluate all cases of disagreement to ensure consensus is achieved.

**Fig 1:**
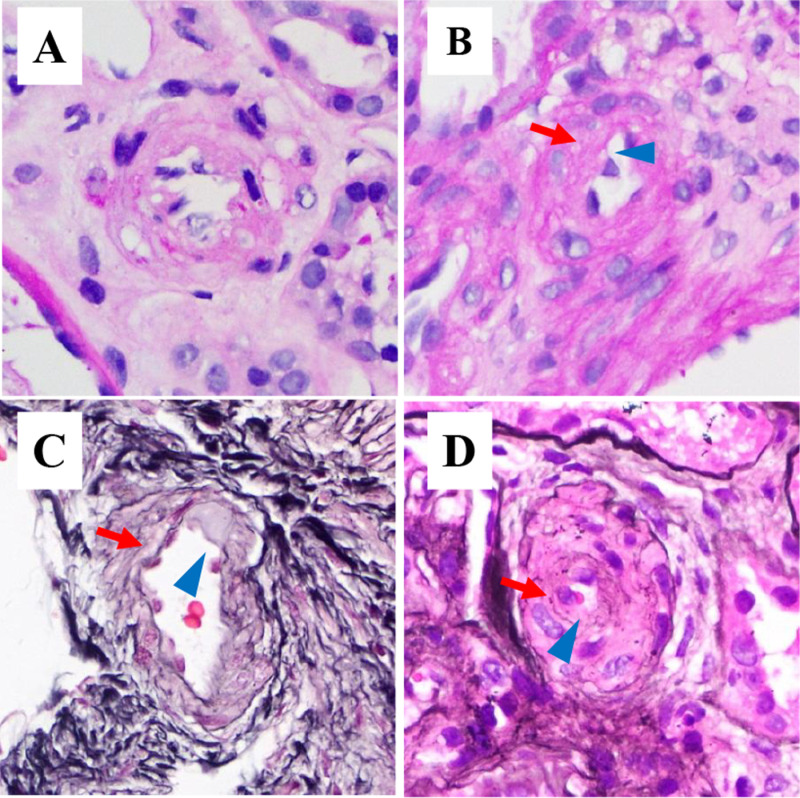
Pathologic picture of chronic arteriolar lesions with different degrees of lesions. (A) Pathological Image of Mild Lesion: slight thickening of the intimal layer and a mild increase in the smooth muscle layers of the media, but no significant luminal stenosis, with luminal stenosis ≤25%.Magnification of 400x, PAS staining. (B) Pathological Image of moderate Lesion:Both the intimal and medial layers are thickened, exhibiting moderate luminal stenosis, with luminal stenosis >26% and <50%.Magnification of 400x, PAS staining. (C) Pathological Image of moderate Lesion: Both the intimal and medial layers are thickened, exhibiting moderate luminal stenosis, with luminal stenosis >26% and <50%, which is accompanied by hyalinization of the vessel wall.Magnification of 400x, PASM staining. (D) Pathological image of severe lesion:thickening of both the intimal and medial layers, a significant increase in the smooth muscle layers of the media, accompanied by severe luminal stenosis, with luminal stenosis >50%, which may or may not be accompanied by hyalinization of the vessel wall.Magnification of 400x, PASM staining.

Chronic arteriolar lesions are defined according to the vascular lesion scoring criteria established in the 2009 Oxford Classification and the Banff 97 grading schema [8,12]. These lesions in the kidneys manifest as arteriolar wall thickening of varying degrees, characterized by mediomuscular hyperplasia, which may or may not be accompanied by intimal thickening, and luminal stenosis, potentially with arteriolar wall hyalinization.Grading criteria for the severity of chronic arteriolar lesions are further delineated as follows, No lesion: The intima shows no significant thickening, the media consists of only 1–2 layers of smooth muscle, and the lumen remains well patent.Mild lesion: There is a slight thickening of the intimal layer and a mild increase in the smooth muscle layers of the media, but no significant luminal stenosis, with luminal stenosis ≤25%(Fig 1A).Moderate lesion: Both the intimal and medial layers are thickened, exhibiting moderate luminal stenosis, with luminal stenosis >26% and <50%, which may or may not be accompanied by hyalinization of the vessel wall (Fig 1B and 1C).Severe lesion: There is marked thickening of both the intimal and medial layers, a significant increase in the smooth muscle layers of the media, accompanied by severe luminal stenosis, with luminal stenosis >50%, which may or may not be accompanied by hyalinization of the vessel wall (Fig 1D).

Evaluation of Intimal thickening and luminal stenosis [12]: Grading of lesions is based on the severity of the most affected vessel’s occlusion.No lesion: No chronic arteriolar intimal thickening.Mild lesion: Arteriolar intimal thickening resulting in up to 25% luminal stenosis.Moderate lesion: Arteriolar intimal thickening resulting in 26% to 50% luminal stenosis.Severe lesion: Severe arteriolar intimal thickening with greater than 50% luminal stenosis.

Evaluation of Arteriolar hyalinosis [15]: the presence of amorphous, homogeneous eosinophilic deposits within the walls of arterioles. Severity grading is not conducted during the assessment. The identification of these specific deposits is sufficient for the diagnosis of moderate chronic arteriolar lesions.

### Defined endpoint and treatments

The initiation of follow-up starts on the day of the renal biopsy. Composite endpoint events are defined as follows: (1)A decline in eGFR by ≥50%, a doubling of serum creatinine, the onset of end-stage renal disease (ESRD), or death. ESRD is characterized by an eGFR less than 15 ml/min/1.73m², requiring either kidney transplantation or maintenance dialysis treatment. Fulfilling any of these criteria constitutes an event. (2)Loss to follow-up. (3)The follow-up cutoff date is December 31, 2022.

The treatment plan adheres to the 2021 KDIGO guidelines [16] and the study conducted by [17–22]:Optimization of supportive therapy, which includes the use of either Angiotensin-converting enzyme inhibitors (ACEI) or angiotensin receptor blockers (ARB).Immunosuppressant therapy: This includes glucocorticoids, immunosuppressants such as cyclophosphamide, mycophenolate mofetil, and tacrolimus.(S1 Table) details the trends in local medication usage and the primary treatment protocols spanning from 2005 to 2021.

### Statistical analysis

Statistical analysis was performed using SPSS 26.0 software. Continuous variables were expressed as mean ± standard deviation for normally distributed variables or median and interquartile range for non-normally distributed variables. Comparisons between groups utilized either independent samples t-test or Mann-Whitney U test for two-group comparisons and a one-way analysis of variance (ANOVA) or the Kruskal-Wallis H test for three-group comparisons, with the Bonferroni test applied for multiple comparisons. Categorical variables were presented as frequency and percentages and analyzed using the χ2 test, Fisher’s exact test, or Kruskal-Wallis test (if applicable). The cumulative incidence of reaching the composite endpoint was assessed using the Kaplan–Meier method and compared using the log-rank test. Univariate and multivariate Cox regression analyses were used to evaluate factors significantly associated with composite endpoints. Multivariable regression analysis was conducted for significant univariate factors, presenting the results as adjusted hazard ratios (aHR) with a 95% confidence interval (CI). Receiver operating characteristic curves (ROC) were plotted, and the area under the curve (AUC) was calculated. The optimal cut-off value was determined based on the maximum Youden index. Logistic regression analysis was utilized to identify the associated risk factors for moderate to severe chronic arteriolar lesions. Missing data were addressed using multiple imputation techniques. Continuous variables were imputed through linear regression models, while categorical variables were imputed using logistic regression models. The imputation process was repeated five times to ensure robustness.All missing variables imputed during the multiple imputation process, along with the covariates included in the regression models, are listed in(S2 Table). A significance level of α=0.05 was used, with P<0.05 considered statistically significant.

## Results

### Differences in demographic and clinicopathologic characteristics of chronic arteriolar lesions of varying degrees

**Fig 2:**
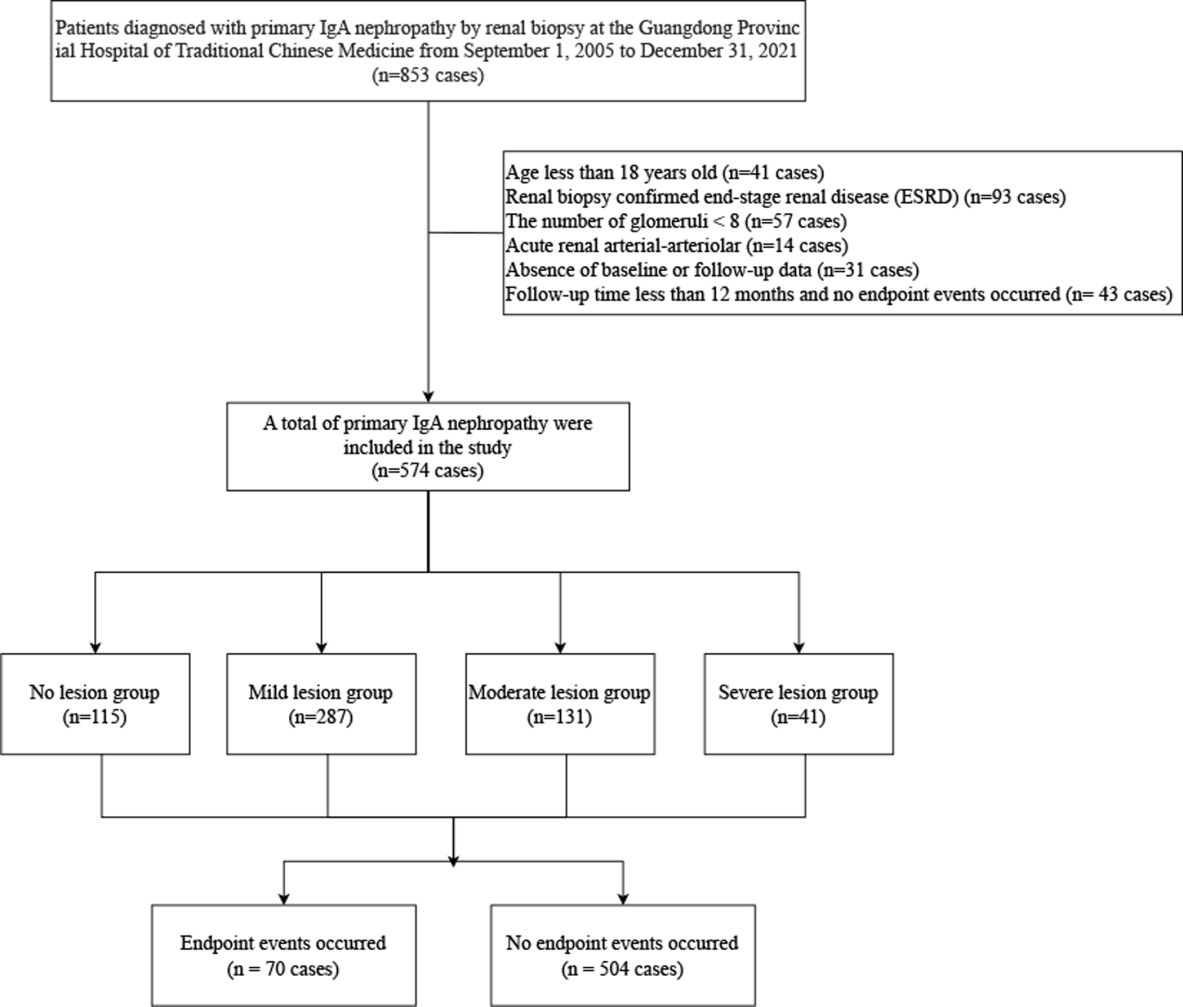
Flowchart of inclusion and follow-up.

This study initially gathered 853 patients diagnosed with primary IgAN. After applying the inclusion criteria, 574 patients met the requirements. The patients’ average age at the time of kidney biopsy was 33 years, with an estimated eGFR of 82.94 ml/min/1.73m². Among them, 115 patients (20.0%) belonged to the no chronic arteriolar lesions group, and 459 patients (80.0%) were categorized into the chronic arteriolar lesions group. Additionally, based on the severity of chronic arteriolar lesions, they were stratified into three groups: mild lesion (287 cases, 62.5%), moderate lesion (131 cases, 28.5%), and severe lesion (41 cases, 8.9%). The median follow-up duration for these patients spanned 53 months, during which the majority received RASi treatment (405 cases, 70.6%), while 239 patients (41.6%) received corticosteroids and/or immunosuppressant treatment. Notably, 70 patients (12.2%) experienced composite endpoint events during the follow-up period (Fig 2).

In the comparison of 574 patients categorized by the degree of chronic arteriolar lesions, it was observed that the moderate and severe groups exhibited higher levels of age (P<0.001), systolic blood pressure (P<0.001), diastolic blood pressure (P<0.001), mean arterial pressure (P<0.001), and creatinine (P<0.001) at the time of renal biopsy. These levels were also elevated compared to both the no lesion group and the mild lesion group. Conversely, eGFR levels were lower (P < 0.001) in the moderate and severe groups in comparison to the no lesion and mild lesion groups. Moreover, the severe lesion group had a higher percentage of hypertensive individuals at the onset of symptoms (14.6% vs 0.0% vs 1.7%, P <0.001) when contrasted with the no lesion and mild lesion groups. Triglyceride levels in the moderate lesion group were notably higher than those in the no lesion and mild lesion groups (1.65 [IQR, 1.16–2.34] mmol/L vs 1.23 [IQR, 0.84–1.72]/ 1.26 [IQR, 0.92–2.03] mmol/L). Similarly, the severe lesion group exhibited higher triglyceride levels than the no lesion group (1.71 [IQR, 1.09–2.73] mmol/L vs 1.23 [IQR, 0.84–1.72] mmol/L), demonstrating statistical significance across the groups (P < 0.001). Fibrinogen levels were found to be elevated in the moderate lesion group, and a statistically significant difference was observed between the moderate disease group and the no lesion group (P=0.002). Furthermore, compared to both the no lesion group and the mild lesion group, a greater number of patients in the moderate lesion group received corticosteroids and/or immunosuppressant treatment (P<0.001), whereas there was no statistically significant difference in treatment between the moderate lesion group and the severe lesion group (Table 1).

**Table 1:** Clinical and pathological characteristics of IgA nephropathy patients with different degree chronic arteriolar lesions.

Variables	No lesion group	Mild lesion group	Moderate lesion group	Severe lesion group	P value
n	115	287	131	41	
Ages (years)	29.00(24.00, 36.00)	31.00(25.00, 41.00)	38.00(31.00, 48.00)^a^^,^^b^	41.00(35.00, 47.00)^a^^,^^b^	< 0.001
Female n (%)	62 (53.9)	146 (50.9)	70 (53.4)	18 (43.9)	0.692
Systolic BP (mmHg)	120.00(110.00, 132.00)	126.00(115.00, 137.00)	132.00(121.00, 145.00)^a^^,^^b^	133.00(127.00, 146.50)^a^^,^^b^	< 0.001
Diastolic BP (mmHg)	76.00(70.00, 86.00)	80.00(72.00, 90.00)^a^	86.00(77.00, 95.00)^a^^,^^b^	88.00(82.50, 95.00)^a^^,^^b^	< 0.001
MAP (mmHg)	92.00(85.33, 99.00)	95.33(86.67, 105.33)^a^	102.67(92.67, 109.67)^a^^,^^b^	104.33(96.67, 110.50)^a^^,^^b^	< 0.001
Hypertension (%)	0(0.0)	5(1.7)	5(3.8)	6(14.6)^a^^,^^b^	< 0.001
History.hypertension (%)	11(9.6)	56(19.5)	53(40.5)^a^^,^^b^	21(51.2)^a^^,^^b^	< 0.001
History.hyperlipidemia (%)	3(2.6)	19(6.6)	12(9.2)	5(12.2)	0.100
Proteinuria(g/d)	0.60(0.36, 1.21)	1.02(0.58, 2.02)^a^	1.57(1.00, 2.98)^a^^,^^b^	0.90(0.53, 1.63)^c^	< 0.001
URBC (per/μl)	39.34(13.00, 96.00)	43.60(20.00, 149.92)	31.00(10.10, 118.80)	20.00(6.50, 43.55)^b^	0.003
BUN (mmol/L)	4.61(3.70, 5.52)	5.17(4.17, 6.36)^a^	6.25(5.17, 8.54)^a^^,^^b^	6.40(5.46, 9.06)^a^^,^^b^	< 0.001
Serum creatinine (μmol/L)	73.00(61.00, 90.00)	87.00(67.00, 109.00)^a^	119.00(92.00, 160.00)^a^^,^^b^	118.00(94.00, 167.60)^a^^,^^b^	< 0.001
eGFR (ml/min/1.73m^2^)	103.96(86.31, 122.91)	87.83(67.68, 111.97)^a^	55.19(41.71,78.37)^a^^,^^b^	57.26(40.31, 82.30)^a^^,^^b^	< 0.001
Uric acid (μmol/L)	365.00(299.00, 440.00)	414.00(319.00, 478.00)	461.00(377.00, 528.00)^a^^,^^b^	459.00(389.00, 554.00)^a^^,^^b^	< 0.001
TP (g/L)	67.50(63.00, 72.00)	66.30(61.00, 71.00)	65.30(58.50, 69.20)^a^	68.20(61.55, 75.95)^c^	0.010
Serum albumin (g/L)	41.40(37.70, 44.50)	39.70(36.30, 43.00)	37.70 (34.00, 41.20)^a^^,^^b^	41.40(36.50, 44.00)	< 0.001
Hemoglobin(g/L)	135.78±19.05	130.09±19.72	126.05±22.22^a^	132.59±23.26	0.003
TC (mmol/L)	4.62(3.95, 5.70)	4.89(4.16, 5.52)	4.82(4.14, 5.85)	4.89(4.44, 5.61)	0.643
LDL-C (mmol/L)	2.93(2.36, 4.21)	3.12(2.49, 3.89)	3.03(2.44, 3.94)	3.18(2.66, 3.92)	0.885
TG (mmol/L)	1.23(0.84, 1.72)	1.26(0.92, 2.03)	1.65(1.16, 2.34)^a^^,^^b^	1.71(1.09, 2.73)^a^	< 0.001
HDL-C (mmol/L)	1.23(1.03, 1.60)	1.19(0.96, 1.45)	1.14(0.92, 1.40)^a^	1.20(0.94, 1.47)	0.029
FIB (g/L)	2.78(2.00, 3.77)	3.06(2.60, 3.86)	3.49(2.70, 4.30)^a^	3.36(2.54, 4.08)	0.002
Oxford Classification(n,%)					
M1	73 (63.5)	268 (93.4)	130 (99.2)	39 (95.1)	<0.001
E1	4 (3.5)	43 (15.0)	26 (19.8)	1 (2.4)	<0.001
S1	32 (27.8)	156 (54.4)	89 (67.9)	25 (61.0)	<0.001
T1/2	2(1.7)/ 0(0.0)	61(21.3)^a^/9(3.1)	52(39.7)^a^^,^^b^/34(26.0)^a^^,^^b^	17(41.5)^a^^,^^b^/10(24.4)^a^^,^^b^	< 0.001
C1/2	18(15.7)/0(0.0)	142(49.5)^a^/19(6.6)^a^	53(40.5)^a^/11(8.4)^a^	13(31.7)/2(4.9)	< 0.001
Globulin.ratio(n,%)					< 0.001
Globulin.ratio25%-50%	4(3.5)	56(19.5)^a^	42(32.1)^a^^,^^b^	18(43.9)^a^^,^^b^	
Globulin.ratio > 50%	0(0.0)	11(3.8)	31(23.7)^a^^,^^b^	7(17.1)^a^^,^^b^	
IgA deposition(n,%)					< 0.001
+	1(0.9)	0(0.0)	1(0.8)	0(0.0)	
2+	25(21.7)	23(8.0)^a^	12(9.2)^a^	12(29.3)^b^^,c^	
3+	85(73.9)	260(90.6)^a^	115(87.8)^a^	28(68.3)^b^^,c^	
4+	4(3.5)	4(1.4)	3(2.3)	1(2.4)	
C3 deposition(n,%)					0.041
–	12(10.4)	9(3.1)	4(3.1)	1(2.4)	
+/-	5(4.3)	4(1.4)	3(2.3)	0(0.0)	
+	9(7.8)	9(3.1)	9(6.9)	2(4.9)	
2+	26(22.6)	60(20.9)	24(18.3)	10(24.4)	
3+	63(54.8)	202(70.4)	90(68.7)	28(68.3)	
4+	0(0.0)	3(1.0)	1(0.8)	0(0.0)	
Corticosteroids.therapy(n,%)	27(23.5)	110(38.3)^a^	60(45.8)^a^	13(31.7)	0.003
Immunosuppressant.therapy(n,%)	9(7.8)	35(12.2)	38(29.0)^a^^,^^b^	7(17.1)	< 0.001
Corticosteroids/immunosuppressant.therapy(n,%)	32(27.8)	118(41.1)	75(57.3)^a^^,^^b^	14(34.1)	< 0.001
RASi.therapy(n,%)	69(60.0)	205(71.4)	99(75.6)	32(78.0)	0.030

BP, blood pressure; MAP, mean arterial pressure; URBC, Urinary red blood cell; HDL-C, high-density lipoprotein cholesterol; LDL-C, low-density lipoprotein cholesterol; eGFR,estimated glomerular filtration rate; BUN, blood urea nitrogen; TP,total serum protein;TG,triglyceride; FIB, fibrinogen; M, mesangial hypercellularity; E, endocapillary hypercellularity; S, segmental glomerulosclerosis; T, tubular atrophy/interstitial fibrosis; C, crescents; IgA deposition, glomerular IgA deposition; C3 deposition, glomerular C3 deposition; RASi, renin-angiotensin system inhibitors.

^a^p value <0.05 compared with the group with no lesion group.

^b^p value *<*0.05 compared with the group with mild lesion group.

^c^p value*<*0.05 compared with the group with moderate lesion group.

In general, when compared to the no lesion group, the chronic arteriolar lesions group exhibited a higher prevalence of M1 (P <0.001) and S1 (P <0.001). Furthermore, the moderate and severe lesion groups demonstrated a greater proportion of T1/2 and glomerulosclerosis ratios between 25% and 50%, as well as above 50%, in contrast to the no lesion and mild lesion groups, with statistically significant intergroup differences (P < 0.001). Additionally, in comparison to the mild lesion and moderate lesion groups, the severe lesion groups displayed a higher percentage of IgA deposition (2+) in the mesangial area (29.3% vs 8.0%/9.2%; P<0.001). The proportion of IgA deposition (3+) was relatively low, and the inter-group comparison remained statistically significant (P < 0.001). Statistical significance was also observed in the comparison of C3 deposition between the groups (P=0.041) (Table 1).

### Prognostic analysis and therapeutic response in chronic arteriolar lesions of varying degrees

**Fig 3:**
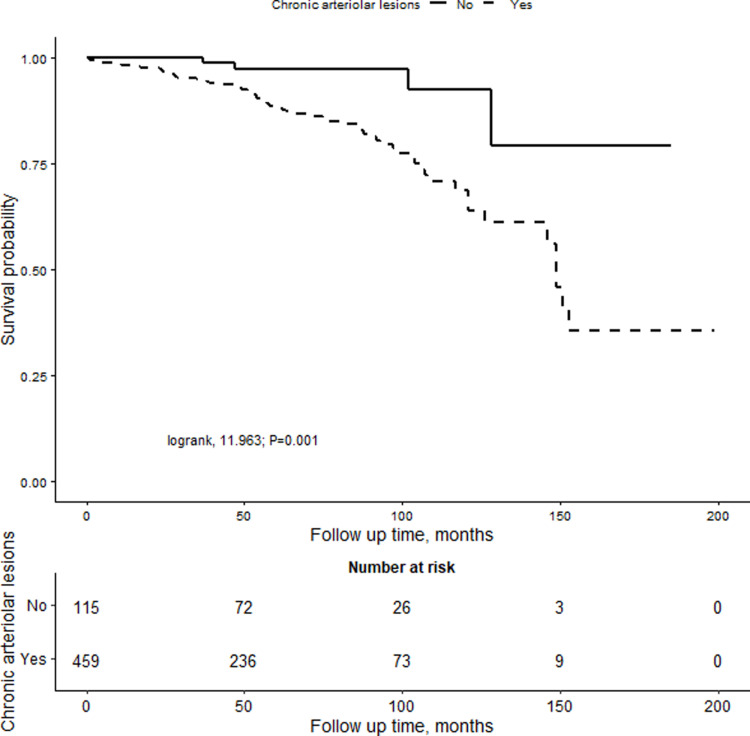
Kaplan-Meier analysis for patients with the presence or absence of chronic arteriolar lesion group.

**Fig 4:**
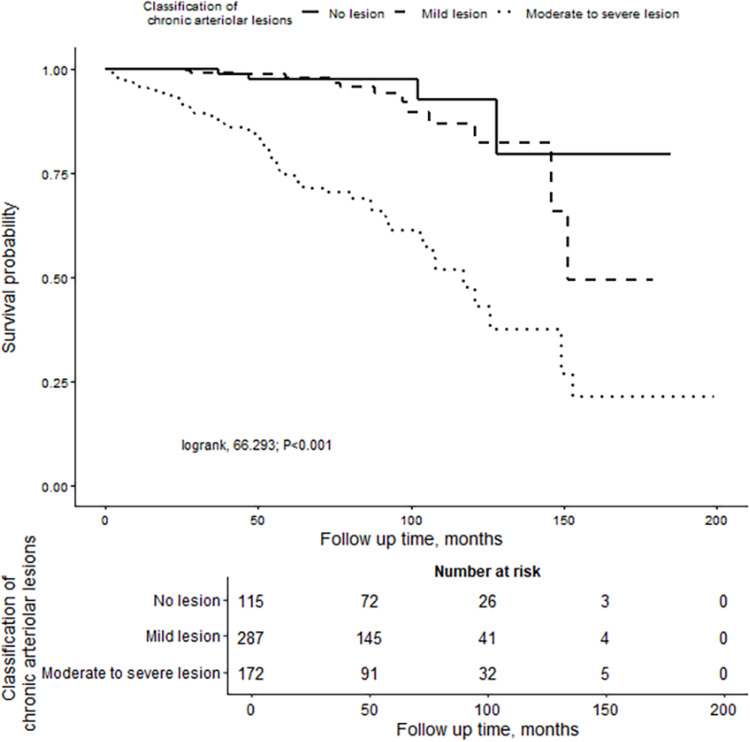
Kaplan-Meier analysis for patients with different degrees of chronic arteriolar lesion group.

In the overall cohort, Kaplan-Meier analysis demonstrated that renal survival time in the chronic arteriolar lesions group was lower than that in the group without chronic arteriolar lesions (log-rank, P = 0.001) (Fig 3). The average cumulative survival time for the moderate to severe lesion group was 113.90±7.66 months, significantly shorter than that of the no lesion group and mild lesion group (log-rank, P < 0.001) (Fig 4). In all patients, The presence or absence of RASi treatment on prognosis was not affected. However, patients treated with corticosteroids and/or immunosuppressants had a shorter renal survival time compared to those not receiving treatment (log-rank, P=0.003) (S1 Fig). Within different degrees of chronic arteriolar lesions lesion subgroups, after adjusting for age, mean arterial pressure (MAP), 24-hour urine protein, creatinine, T lesion, and C lesion, it was observed that the renal survival time in the moderate to severe lesion group treated with RASi was significantly longer than that in the untreated group (aHR=0.429, 95%CI: 0.194–0.948; P=0.036) (S2 and S3 Figs).

Univariate Cox regression analysis indicated that mean arterial pressure, urinary protein quantification, blood creatinine, eGFR, blood urea nitrogen, blood uric acid, triglycerides, fibrinogen, S1, T1/2, C2, glomerular sclerosis ratio of 25%-50%, and >50%, as well as moderate to severe chronic arteriolar lesions lesions (HR=9.959, 95% CI: 3.602–27.534; P<0.001), and corticosteroids and/or immunosuppressant treatment were risk factors influencing composite endpoint events (P<0.05)(Table 2). Multivariable Cox regression analysis revealed that moderate to severe chronic arteriolar lesions (aHR=3.357, 95%CI: 1.018–11.071; P=0.047), creatinine, E1, S1, T2, and C2 were independent risk factors for composite endpoint events (P<0.05) (Table 3).

**Table 2:** Cox Univariate Regression Analysis: Risk Factors for Adverse Renal Outcomes.

Variables	HR	95% CI	P value
Female n (%)	1.481	0.908 - 2.416	0.116
Ages (years)	1.007	0.985 - 1.029	0.531
MAP (mmHg)	1.024	1.009 - 1.040	0.002
History.hypertension (%)	2.209	1.371 - 3.558	0.001
Proteinuria(g/d)	1.149	1.080 - 1.222	< 0.001
URBC (per/μl)	1.000	1.000 - 1.000	0.189
BUN (mmol/L)	1.248	1.189 - 1.309	< 0.001
Serum creatinine (μmol/L)	1.020	1.017 - 1.023	< 0.001
eGFR (ml/min/1.73m^2^)	0.959	0.949 - 0.970	< 0.001
Uric acid (μmol/L)	1.006	1.004 - 1.008	< 0.001
TG (mmol/L)	1.191	1.054 - 1.346	0.005
FIB(g/L)	1.375	1.162 - 1.627	< 0.001
M1	6.560	0.909 - 47.331	0.062
E1	0.758	0.386 - 1.487	0.420
S1	3.961	2.025 - 7.949	< 0.001
T1	2.423	1.277 - 4.598	0.007
T2	23.124	12.832 - 41.671	< 0.001
C1	1.039	0.617 - 1.749	0.885
C2	3.932	1.738 - 8.896	0.001
Globulin.ratio25%-50%	4.132	2.331- 7.324	< 0.001
Globulin.ratio > 50%	12.119	6.776 - 21.675	< 0.001
Mild lesion, n (%)	1.624	0.529 - 4.985	0.396
Moderate lesion, n (%)	9.798	3.517 - 27.297	< 0.001
Severe lesion, n (%)	10.812	3.315 - 35.260	< 0.001
Moderate to severe lesion, n (%)	9.959	3.602 - 27.534	< 0.001
Corticosteroids.therapy(%)	2.221	1.383 - 3.568	0.001
Immunosuppressant.therapy(%)	1.839	1.083 - 3.120	0.024
Corticosteroids/immunosuppressant.therapy(%)	2.022	1.250- 3.272	0.004
RASi.therapy(%)	0.831	0.498 - 1.388	0.480

CI, confidence interval. *p* < 0.05 was considered significant.

**Table 3:** Cox Multivariate Regression Analysis: Independent Risk Factors for Adverse Renal Outcomes.

Variables	Model 1			Model 2			Model 3		
	aHR	95% CI	P value	aHR	95% CI	P value	aHR	95% CI	P value
Ages (years)	0.998	0.973-1.024	0.875	0.995	0.969 -1.022	0.730	0.997	0.970-1.024	0.801
MAP (mmHg)	0.992	0.973 -1.011	0.413	0.990	0.971-1.009	0.303	0.992	0.973-1.011	0.411
Proteinuria(g/d)	1.033	0.925 -1.153	0.565	1.042	0.925-1.173	0.500	1.030	0.905-1.173	0.652
Serum creatinine (μmol/L)	1.015	1.009 -1.021	< 0.001	1.014	1.008-1.021	< 0.001	1.014	1.008-1.020	< 0.001
Uric acid (μmol/L)	1.000	0.997 -1.003	0.788	0.999	0.996-1.002	0.704	0.999	0.996-1.002	0.624
TG(mmol/L)	1.081	0.903 -1.293	0.397	1.044	0.863-1.263	0.658	1.034	0.853-1.255	0.732
M1	2.283	0.301 -17.286	0.424	1.553	0.190 -12.722	0.681	1.433	0.173-11.856	0.739
E1	0.409	0.180 -0.929	0.033	0.356	0.154-0.827	0.016	0.373	0.159-0.875	0.023
S1	2.213	1.057 -4.632	0.035	2.150	1.012 -4.568	0.046	2.208	1.024- 4.761	0.043
T1	1.374	0.660 -2.862	0.396	0.851	0.390-1.861	0.687	0.995	0.450- 2.201	0.990
T2	4.601	1.918 -11.038	0.001	2.716	1.085 -6.800	0.033	3.185	1.266-8.015	0.014
C1	0.991	0.530 -1.855	0.978	1.057	0.559-2.000	0.864	1.034	0.537-1.991	0.920
C2	2.595	1.061 -6.346	0.037	2.831	1.136-7.059	0.026	3.250	1.273-8.293	0.014
Mild lesion, n (%)	NA	NA	NA	1.078	0.330 -3.521	0.901	1.026	0.310-3.393	0.967
Moderate to severe lesion, n (%)	NA	NA	NA	3.336	1.020 -10.916	0.046	3.357	1.018-11.071	0.047
Corticosteroids/immunosuppressant.therapy(%)	NA	NA	NA	NA	NA	NA	0.803	0.437 -1.474	0.479
RASi.therapy(%)	NA	NA	NA	NA	NA	NA	0.584	0.320-1.066	0.080

aHR, adjusted Hazard Ratio; CI, confidence interval.

p < 0.05 was considered significant.

Model 1: Including Ages, MAP, Proteinuria, Serum creatinine, Uric acid, TG, MEST-C score.

Model 2: Including Model 1 + grouping according to the degree of chronic arteriolar lesions.

Model 3: Including Model 2 + Corticosteroids/immunosuppressant. therapy, RASi treatment.

### Triglycerides and fibrinogen are associated with the progression of moderate to severe chronic arteriolar lesions

**Fig 5:**
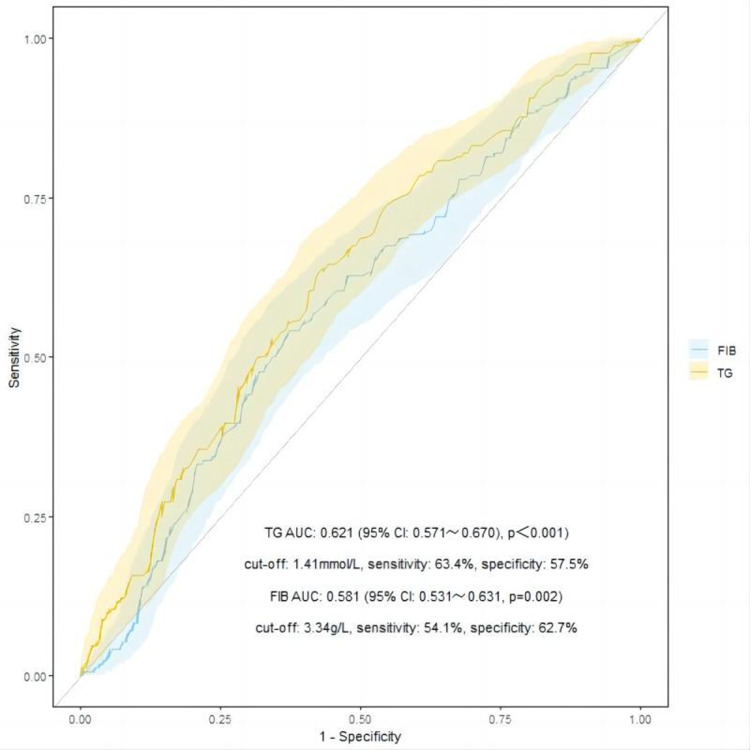
ROC curves of subjects with TG and FIB as test variables and the moderate to severe chronic arteriolar lesions as outcome variables.

In comparison to the no lesions group and mild lesions group, the moderate to severe lesions group exhibited significantly higher levels of triglycerides and fibrinogen (P < 0.05). ROC curves were plotted, and the Youden index was calculated, resulting in optimal cut-off values of 1.41 mmol/L for triglycerides and 3.34 g/L for fibrinogen (Fig 5).

Multivariable logistic regression analysis identified “age, creatinine (OR=1.007, 95% CI: 1.001-1.013; P=0.016), M1 lesions, and T1/2 lesions” as independent risk factors for the development of moderate to severe chronic arteriolar lesions (P<0.05) (Table 4). Cox multivariable regression analysis revealed that creatinine (aHR=1.022, 95% CI: 1.013–1.030; P=0.001), T2, and C2 were independent risk factors for the occurrence of composite endpoint events in patients with moderate to severe chronic arteriolar lesions. Notably, RASi treatment was identified as a protective factor against composite endpoint events (P<0.05) (Table 5).

**Table 4:** Multivariable Logistic Regression Analysis: Risk Factors for Moderate to Severe Chronic Arteriolar Lesions.

Variables	OR	95% CI	P value
Ages (years)	1.052	1.031- 1.073	< 0.001
Serum creatinine (μmol/L)	1.007	1.001- 1.013	0.016
TG≥1.41(mmol/L)	1.707	1.091-2.669	0.019
M1	4.918	1.452- 16.656	0.010
T1	4.029	2.446-6.639	< 0.001
T2	13.246	5.078-34.552	< 0.001

CI, confidence interval. *p* < 0.05 was considered significant.

**Table 5:** Cox multivariate regression analysis of patients with moderate to severe chronic arteriolar lesions.

Variables	aHR	95% CI	P value
Female n (%)	1.079	0.470-2.481	0.857
Ages (years)	1.031	0.988-1.076	0.158
MAP (mmHg)	1.008	0.985-1.031	0.497
Proteinuria(g/d)	0.900	0.760-1.065	0.220
Serum creatinine (μmol/L)	1.022	1.013-1.030	0.001
Uric acid (μmol/L)	0.997	0.993-1.001	0.170
TG≥1.41(mmol/L)	4.317	1.505-12.380	0.007
FIB≥3.34(g/L)	2.280	1.191-4.365	0.013
E1	0.347	0.107-1.127	0.078
S1	1.360	0.491-3.763	0.554
T1	3.225	0.821-12.666	0.093
T2	11.905	2.651-53.457	0.001
C1	1.823	0.810-4.103	0.147
C2	5.316	1.421-19.884	0.013
Corticosteroids/immunosuppressant.therapy(%)	0.842	0.352-2.012	0.699
RASi.therapy(%)	0.290	0.112-0.752	0.011

aHR, adjusted Hazard Ratio; CI, confidence interval.

*p* < 0.05 was considered significant.

## Discussion

This study identified significant disparities in clinical, pathological, and prognostic aspects across various degrees of chronic arteriolar lesions lesions. Notably, the moderate to severe chronic arteriolar lesions group exhibited a strong association with IgAN progression. In comparison to other lesion groups, the moderate to severe chronic arteriolar lesions group displayed more severe indicators such as elevated creatinine levels, and increased T1/2 lesions. These factors emerged as independent risk factors for unfavorable renal outcomes. In this study, the moderate to severe lesion group was characterized by thickening of intimal and medial layers with or without accompanied by arteriolar hyalinosis. Prior research has proposed a potential mechanism through which chronic arteriolar lesions lesions may contribute to IgAN progression, involving hypoxia. Hypoxia can expedite renal artery injury, glomerulosclerosis, and interstitial fibrosis, culminating in adverse renal outcomes [23]. Another study has suggested that arteriolar hyalinosis might serve as an indicator of reduced interstitial blood flow and hypoxia in glomerulonephritis [24]. Consequently, the moderate to severe lesion group in our study presented more unfavorable clinical and pathological indicators, indicating that the combination of moderate to severe arteriolar hyalinosis with intimal thickening might be linked to a poorer renal prognosis.

Age is a significant risk factor for the development of chronic arteriolar lesions in patients with IgAN (OR = 1.052, 95% CI: 1.031–1.073; P < 0.001), consistent with previous research findings [7,25],This suggests that vascular lesions’ frequency and severity increase with age. Age-related renal vascular changes may be linked to reduced angiogenic factors such as vascular endothelial growth factor (VEGF) and an increase in anti-angiogenic factors [26].

Earlier studies have established a close association between blood pressure and arteriolar hyalinosis [27]. Our findings revealed that as the degree of chronic arteriolar lesions increased, blood pressure gradually elevated in IgAN patients, while eGFR declined progressively. This phenomenon could be attributed to the worsening vascular lesions in IgAN, leading to excessive activation of the renin-angiotensin-aldosterone system (RAAS), which in turn resulted in a continuous rise in blood pressure [28]. A study by Sugiura et al. [29] suggested that the use of RASi treatment at any point during the follow-up period might effectively slow down the disease progression in IgAN patients with concurrent chronic arteriolar lesions. In our study, a majority of patients received RASi treatment (405 cases, 70.6%), and renal survival time was significantly longer in the moderate to severe lesion group that received RASi therapy compared to the untreated group (aHR = 0.429, 95% CI: 0.194–0.948, P = 0.036). Multivariate Cox regression analysis of the moderate to severe lesion groups indicated that RASi treatment acted as a protective factor against adverse renal outcomes (aHR=0.290, 95%CI: 0.112–0.752, P=0.011). This could be attributed to the more severe intimal thickening and arteriolar hyalinosis in the moderate to severe lesion group. Therefore, supportive therapy may potentially inhibit RAAS activation and improve renal prognosis in IgAN patients, particularly when chronic lesions are more severe.

Serum creatinine is a well-established marker for assessing the severity of renal disease, with elevated levels typically indicating a poor prognosis. Ikee et al. [30] found through univariate analysis that IgAN patients with elevated serum creatinine primarily exhibit renal arteriosclerosis, luminal stenosis, and significant vascular wall thickening, which are associated with worse outcomes. In this study, multivariate logistic regression analysis revealed a significant association between serum creatinine and moderate-to-severe chronic arteriolar lesions (OR = 1.007, 95% CI: 1.001–1.013; P = 0.016; P < 0.05), suggesting that impaired renal function may be one of the driving factors for the progression of moderate-to-severe chronic arteriolar lesions. However, since this study was based solely on a single measurement of serum creatinine at the time of renal biopsy, it was not possible to establish a causal relationship between elevated serum creatinine and moderate-to-severe chronic arteriolar lesions. Future studies are needed to elucidate the dynamic interplay between these two factors using multiple follow-up data points.

This study demonstrates that T lesions are significantly associated with the progression of moderate to severe chronic arteriolar lesions(T1, OR=4.029, 95% CI: 2.446–6.639, P<0.001; T2, OR=13.246, 95% CI: 5.078–34.552, P<0.001), a finding consistent with previous research [13,29]. Naesens et al. highlighted that T lesions are strongly associated with other chronic injuries, such as arteriolar hyalinosis and intimal thickening [31]. Furthermore, Bazzi et al. suggested that the primary mechanism behind tubulointerstitial damage is hemodynamic changes leading to decreased interstitial blood flow, with arteriolar hyalinosis being considered a potential surrogate marker for T lesions [24].

In this study, we observed that the proportion of glomerular IgA deposition (2+) was significantly higher in the severe lesion group compared to the mild and moderate lesion groups (29.3% vs 8.0%/9.2%; P<0.001). According to Monteiro et al., the pathogenesis may involve the deposition of IgA and other immune complexes within the walls of small arteries and arterioles, triggering the release of inflammatory mediators and cytokines [32]. This inflammatory response promotes smooth muscle cell proliferation and endothelial damage, ultimately leading to vascular immunological injury. However, our study did not find a significant association between IgA deposits and moderate to severe chronic arteriolar lesions, which may be due to the relatively small number of patients with severe chronic arteriolar lesions (41 cases, 8.9%). Further studies with larger sample sizes are needed to confirm this association.

Elevated plasma triglyceride levels have been independently associated with the progression of human renal diseases [33]. Previous studies have established a correlation between triglycerides and arteriolar hyalinosis [27]. The accumulation of triglycerides in the kidneys may lead to lipid nephrotoxicity, triggering podocyte detachment, apoptosis, mesangial cell proliferation, and glomerular lesions, thereby accelerating renal damage [34–36]. Consequently, triglycerides are closely linked with severe pathological damage and poor prognosis in IgAN [25,37,38]. However, before this study, no research had addressed the optimal range for controlling triglyceride levels in IgAN. Our study constructed a ROC curve and calculated the Youden index to identify the optimal cutoff value for triglycerides at 1.41 mmol/L. Elevated serum fibrinogen levels contribute to the development of small artery disease by increasing blood viscosity, stimulating fibrin formation, and enhancing platelet aggregation [39]. Similarly, the optimal cut-off value for fibrinogen was determined to be 3.34 g/L. Further analysis revealed that TG ≥ 1.41 mmol/L and FIB ≥ 3.34 g/L are significantly associated with the occurrence of composite endpoints of moderate to severe chronic arteriolar lesions (aHR=4.317, 95% CI 1.505–12.380, p=0.007; aHR=2.280, 95% CI 1.191–4.365, p=0.013). Despite the statistical significance of the associations between triglycerides, fibrinogen, and moderate to severe chronic arteriolar lesions, the discriminative power of these biomarkers was limited, with AUC values of 0.621 for triglycerides (95% CI 0.571–0.670, p<0.001) and 0.581 for fibrinogen (95% CI 0.531–0.631, p=0.002). These results may be attributed to a mismatch between the model and the current data. Additionally, as this study was cross-sectional, it is impossible to establish causal relationships. The sensitivity and specificity of the triglyceride and fibrinogen cut-off values within the normal range were relatively low, limiting their utility in clinical practice. Future studies should employ prospective designs, select models better suited to the data, and incorporate longitudinal data analysis to validate the reliability of these biomarkers and their potential applicability in clinical practice.

This innovative approach which categorizes different degrees of chronic arteriolar lesions, fulfills a clinical need. This classification method can better guide clinicians in providing treatment recommendations for IgAN, thereby maximizing improvements in long-term kidney survival rates for patients.

However, our study does have its limitations. Firstly, it was a retrospective study with a relatively small sample size, and there were relatively few severe cases included (n=41). As this study is based on cross-sectional data, it only reflects the static situation at the time of the study, which presents certain limitations.The follow-up duration was relatively short compared to the progression rate of IgAN. Additionally, this study was conducted at a single center, and the study population predominantly consisted of East Asians, potentially introducing biases related to race, geographic factors, etc., which limits its external generalizability. Therefore, it is imperative to conduct multicenter, large-sample prospective clinical studies to validate and reinforce these findings.

## Conclusion

Analyzing IgAN patients with different degrees of chronic arteriolar lesions group revealed that moderate-to-severe chronic arteriolar lesions were independent risk factors for adverse renal prognosis in IgAN. The results of this study have a high degree of accuracy, differentiation, and predictive ability, suggesting its potential utility for clinical diagnosis, medical intervention, and prognostic judgment in patients with IgAN.

## Supporting information

S1 Table Trends in Medication Practice Evolution and Principal Treatment Regimens from 2005 to 2021. (DOCX)

S2 Table Variables with Multiple Imputation for Missing Data and Covariates Included in the Imputation Regression Models. (DOCX)

S1 Fig Kaplan-Meier analysis for patients with or without Corticosteroids and/or immunosuppressant therapy. (TIF)

S2 Fig Kaplan-Meier analysis for the moderate-to-severe lesion group with and without RASi treatment, After adjusting for age, MAP, 24h urine protein, serum creatinine, T lesion, C lesion. (TIF)

S3 Fig Forest plot with subgroup analyses of the moderate-to-severe lesion group and RASi treatment. (TIF)
