# Supplementary material for: Moderate to severe chronic arteriolar lesions is an independent risk factor for adverse renal outcomes in IgA nephropathy
Source: PLoS One. 2025 Apr 24;20(4):e0320635. doi: 10.1371/journal.pone.0320635 (PMC12021281; doi:10.1371/journal.pone.0320635)
Supplement: S1 Table — (DOCX) [file pone.0320635.s001.docx]

**S1 Table：Trends in Medication Practice Evolution and Principal Treatment Regimens from 2005 to 2021**

| Treatment Protocol | Therapeutic Regimen | |
| --- | --- | --- |
| Supportive therapy  （ACEI/ARB） | 2005-2021 | When urinary protein exceeds 1g/d, it is  recommended to initiate treatment with a gradually increasing dosage up to the maximum tolerated dose |
|  | 2021-Endpoint | When urinary protein exceeds 0.5 g/d, it is  recommended to initiate treatment with a gradually increasing dosage up to the maximum tolerated dose |
| Glucocorticoid therapy | **Adequate Glucocorticoid Therapy**: Prednisolone 60 mg or  Methylprednisolone 48 mg, with a gradual tapering schedule.  **Application**: Indicated for IgA nephropathy with nephrotic syndrome, encompassing conditions such as proteinuria and rapidly progressive  renal function deterioration. | |
|  | **Low-Dose** glucocorticoid therapy**:**Prednisolone acetate 30 mg/d and  methylprednisolone 24 mg/d, with a gradual tapering schedule.  **Application Scope:**For patients whose urinary protein remains >1 g  after 3-6 months of supportive therapy. | |
| Immunosuppressants  combined with low-  dose glucocorticoids | Dosage of Mycophenolate Mofetil（MMF）/Cyclophosphamide Combined with Glucocorticoids for Treatment：  （1）MMF, 1.5 g/d, for 6 months and prednisone, 0.4 to  0.6 mg/kg/d, for 2 months and then tapered by 20% per month for  the next 4 months;   1. Patients were administered cyclophosphamide continuously for   three months with a daily dosage of 1.5 mg/kg. Subsequently,  azathioprine was introduced from the fourth through the thirty-sixth month at a daily dosage of 1.5 mg/kg. Oral prednisone was initiated at 40 mg daily, tapered gradually to 10 mg daily during the initial three months, maintained at 10 mg daily from the fourth to the sixth month, and reduced to 7.5 mg daily from the seventh to the thirty-sixth month.  Applications: IgAN characterized by rapidly deteriorating  renal function with crescentic lesions; active proliferative IgAN; rapidly progressive glomerulonephritis type IgAN. | |

ACEI,angiotensin-converting enzyme inhibitors; ARB,angiotensin receptor blockers.
