# Supplementary material for: Moderate to severe chronic arteriolar lesions is an independent risk factor for adverse renal outcomes in IgA nephropathy
Source: PLoS One. 2025 Apr 24;20(4):e0320635. doi: 10.1371/journal.pone.0320635 (PMC12021281; doi:10.1371/journal.pone.0320635)
Supplement: S2 Table — (DOCX) [file pone.0320635.s002.docx]

**S2 Table：**Variables with Multiple Imputation for Missing Data and Covariates Included in the Imputation Regression Models****

| **Missing** Variables | Missing Rate（%） | Included Covariates |
| --- | --- | --- |
| Hb | 13.9 | Age、Sex、Body Mass Index、History of hypertension、Diabetes history、Cardiovascular history、History of hyperlipidemia、Hb、Platelet、URBC、Scr、BUN、UA、TC、LDL-C、TG、HDL-C、FIB、The Oxford classification (MEST-C scoring) . |
| URBC | 2.0 |  |
| UA | 3.9 |  |
| TC | 1.8 |  |
| LDL-C | 2.9 |  |
| TG | 2.0 |  |
| HDL-C | 2.9 |  |
| PT | 28.7 |  |
| PTA | 29.0 |  |
| INR | 29.3 |  |
| FIB | 19.3 |  |
| APTT | 27.3 |  |
| Glomerular IgA deposition | 2.2 |  |
| Glomerular C3 deposition | 2.2 |  |

PT，Prothrombin Time；PTA，Prothrombin Activity ；INR，International Normalized Ratio；APTT,Activated Partial Thromboplastin Time.
