# Supplementary figures and images for: Moderate to severe chronic arteriolar lesions is an independent risk factor for adverse renal outcomes in IgA nephropathy
Source: PLoS One. 2025 Apr 24;20(4):e0320635. doi: 10.1371/journal.pone.0320635 (PMC12021281; doi:10.1371/journal.pone.0320635)

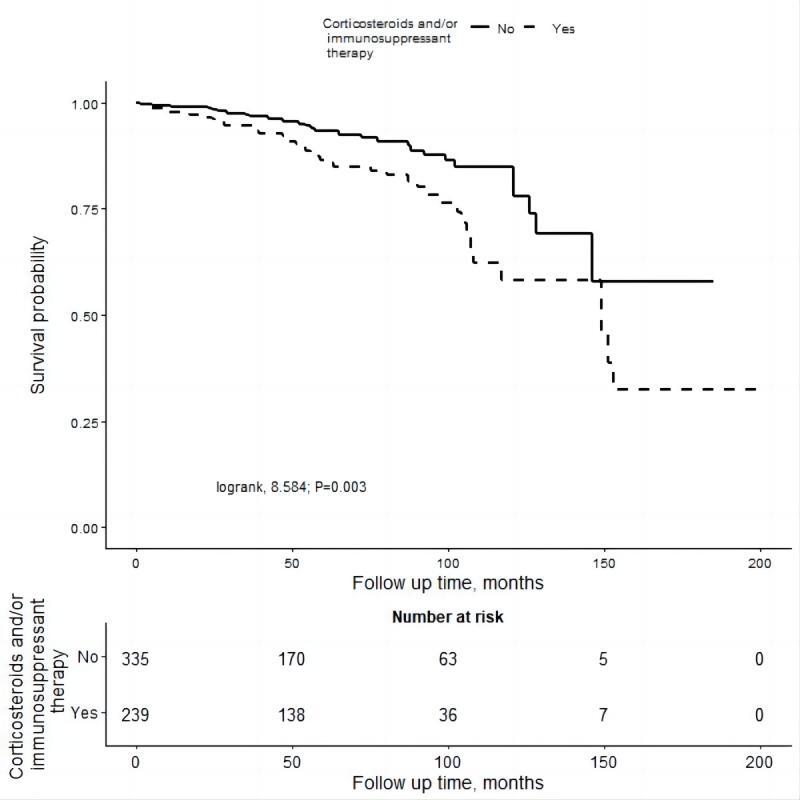

Supplement: S1 Fig — (TIF) [file pone.0320635.s003.tif]

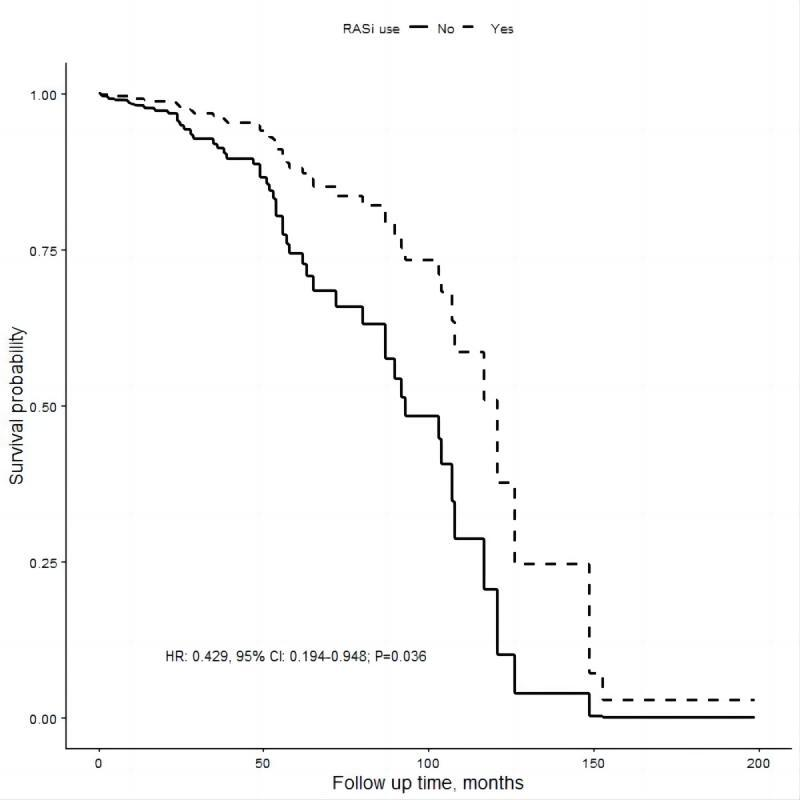

Supplement: S2 Fig — (TIF) [file pone.0320635.s004.tif]

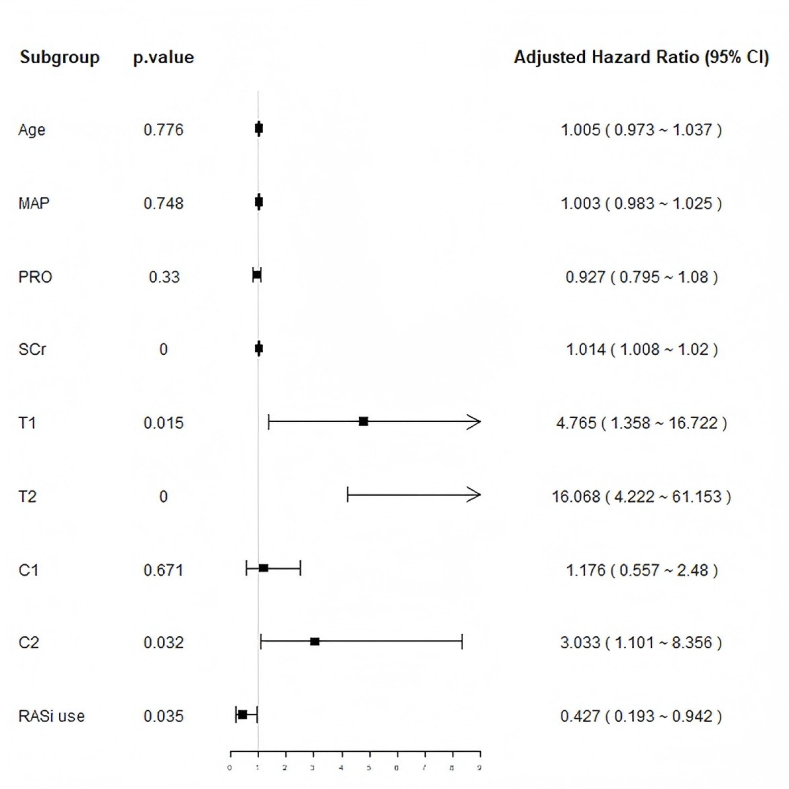

Supplement: S3 Fig — (TIF) [file pone.0320635.s005.tif]
